# Nonlinear Craig Interpolant Generation

**DOI:** 10.1007/978-3-030-53288-8_20

**Published:** 2020-06-13

**Authors:** Ting Gan, Bican Xia, Bai Xue, Naijun Zhan, Liyun Dai

**Affiliations:** 8 Microsoft Research Lab, Redmond, WA USA; 9 University of Southern California, Los Angeles, CA USA; 10 School of Computer Science, Wuhan University, Wuhan, China; 11 LMAM, School of Mathematical Sciences, Peking University, Beijing, China; 12 State Key Laboratory of Computer Science, Institute of Software, CAS, Beijing, China; 13 University of Chinese Academy of Sciences, Beijing, China; 14 RISE, School of Computer and Information Science, Southwest University, Chongqing, China

**Keywords:** Craig interpolant, Archimedean condition, Semi-definite programming, Program verification, Sum of squares

## Abstract

Craig interpolant generation for non-linear theory and its combination with other theories are still in infancy, although interpolation-based techniques have become popular in the verification of programs and hybrid systems where non-linear expressions are very common. In this paper, we first prove that a polynomial interpolant of the form $h(\mathbf {x})>0$ exists for two mutually contradictory polynomial formulas $\phi (\mathbf {x},\mathbf {y})$ and $\psi (\mathbf {x},\mathbf {z})$, with the form $f_1\ge 0\wedge \cdots \wedge f_n\ge 0$, where $f_i$ are polynomials in $\mathbf {x},\mathbf {y}$ or $\mathbf {x},\mathbf {z}$, and the quadratic module generated by $f_i$ is Archimedean. Then, we show that synthesizing such interpolant can be reduced to solving a semi-definite programming problem ($\mathrm{SDP}$). In addition, we propose a verification approach to assure the validity of the synthesized interpolant and consequently avoid the unsoundness caused by numerical error in $\mathrm{SDP}$ solving. Besides, we discuss how to generalize our approach to general semi-algebraic formulas. Finally, as an application, we demonstrate how to apply our approach to invariant generation in program verification.

## Introduction

Interpolation-based techniques have become popular in recent years because of their inherently modular and local reasoning, which can scale up existing formal verification techniques like theorem proving, model-checking, abstract interpretation, and so on, while the scalability is the bottleneck of these techniques. The study of interpolation was pioneered by Kraj$\mathrm{\acute{i}\breve{c}}$ek [20] and Pudlák [30] in connection with theorem proving, by McMillan in connection with model-checking [25], by Graf and Saïdi [14], Henzinger *et al.* [16] and McMillan [26] in connection with abstraction like CEGAR, by Wang *et al.* [17] in connection with machine-learning based program verification.

Craig interpolant generation plays a central role in interpolation-based techniques, and therefore has drawn increasing attention. In the literature, there are various efficient algorithms proposed for automatically synthesizing interpolants for decidable fragments of first-order logic, linear arithmetic, array logic, equality logic with uninterpreted functions (EUF), etc., and their combinations, and their use in verification, e.g., [6, 16, 18, 19, 26, 27, 33, 33, 37] and the references therein. Additionally, how to compare the strength of different interpolants is investigated in [9]. However, interpolant generation for non-linear theory and its combination with the aforementioned theories is still in infancy, although nonlinear polynomials inequalities are quite common in safety-critical software and embedded systems [38, 39].

In [7], Dai *et al.* had a first try and gave an algorithm for generating interpolants for conjunctions of mutually contradictory nonlinear polynomial inequalities based on the existence of a witness guaranteed by Stengle’s Positivstellensatz [36], which is computable using semi-definite programming ($\mathrm{SDP}$). Their algorithm is incomplete in general but if all variables are bounded (called Archimedean condition), then it becomes complete. A major limitation of their work is that two mutually contradictory formulas $\phi$ and $\psi$ must have the same set of variables. In [10], Gan *et al.* proposed an algorithm to generate interpolants for quadratic polynomial inequalities. The basic idea is based on the insight that for analyzing the solution space of concave quadratic polynomial inequalities, it suffices to linearize them by proving a generalization of Motzkin’s transposition theorem for concave quadratic polynomial inequalities. Moreover, they also discussed how to generate interpolants for the combination of the theory of quadratic concave polynomial inequalities and *EUF* based on the hierarchical calculus proposed in [34] and used in [33]. Obviously, *quadratic concave* polynomial inequalities is a very restrictive class of polynomial formulas, although most of existing abstract domains fall within it as argued in [10]. Meanwhile, in [13], Gao and Zufferey presented an approach to extract interpolants for non-linear formulas possibly containing transcendental functions and differential equations from proofs of unsatisfiability generated by $\delta$-decision procedure [12] based on interval constraint propagation (ICP) [1] by transforming proof traces from $\delta$-complete decision procedures into interpolants that consist of Boolean combinations of linear constraints. Thus, their approach can only find the interpolants between two formulas whenever their conjunction is not $\delta$-satisfiable. Similar idea was also reported in [21]. In [5], Chen *et al.* proposed an approach for synthesizing non-linear interpolants based on counterexample-guided and machine-learning, but it relies on quantifier elimination in order to guarantee the completeness and convergence, which gives rise to the low efficiency of their approach theoretically. In [35], Srikanth *et al.* presented an approach called *CAMPY* to exploit non-linear interpolant generation, which is achieved by abstracting non-linear formulas (possibly with non-polynomial expressions) to the theory of linear arithmetic with uninterpreted functions, i.e., EUFLIA, to prove and/or disprove if a given program satisfies a given property, that may contain nonlinear expressions.

### Example 1

In order to compare the approach proposed in this paper and the ones aforementioned, consider

$$\begin{aligned} \phi= & {} -2xy^2 +x^2 - 3xz-y^2 -yz+z^2-1\ge 0 \wedge 100-x^2-y^2\ge 0 \wedge \\&x^2z^2 +y^2z^2-x^2-y^2+ \frac{1}{6} (x^4+2x^2y^2+y^4)-\frac{1}{120}(x^6+y^6)-4 \le 0; \\ \psi= & {} 4(x-y)^4 +(x+y)^2 +w^2-133.097\le 0 \wedge 100(x+y)^2-w^2(x-y)^4-3000 \ge 0. \end{aligned}$$

It can be checked that $\phi \wedge \psi \models \bot$.

**Fig. 1.**
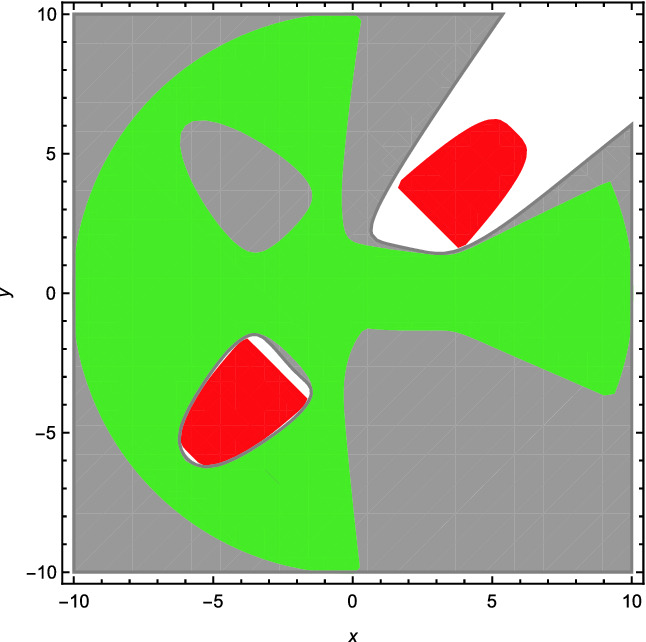
Example 1. (Green region: the projection of $\phi (x,y,z)$ onto *x* and *y*; red region: the projection of $\psi (x,y,w)$ onto *x* and *y*; gray region plus the green region: the synthesized interpolant $\{(x,y)\mid h(x,y)>0\}$.) (Color figure online)

Obviously, synthesizing interpolants for $\phi$ and $\psi$ in this example is beyond the ability of the above approaches reported in [7, 10]. Using the method in [13] implemented in dReal3 it would return “SAT” with $\delta =0.001$, i.e., $\phi \wedge \psi$ is $\delta$-satisfiable, and hence it cannot synthesize any interpolant using [12]’s approach with any precision greater than 0.001^1^. While, using our method, an interpolant $h>0$ with degree 10 can be found as shown in Fig. 1,^2^. Additionally, using the symbolic procedure REDUCE, it can be proved that $h>0$ is indeed an interpolant of $\phi$ and $\psi$.

In this paper, we investigate this issue and consider how to synthesize an interpolant for two polynomial formulas $\phi (\mathbf {x},\mathbf {y})$ and $\psi (\mathbf {x},\mathbf {z})$ with $\phi (\mathbf {x},\mathbf {y})\wedge \psi (\mathbf {x},\mathbf {z}) \models \bot$, where

$$\phi (\mathbf {x},\mathbf {y}): f_1(\mathbf {x},\mathbf {y}) \ge 0 \wedge \cdots \wedge f_m(\mathbf {x},\mathbf {y})\ge 0$$, $$\psi (\mathbf {x},\mathbf {z}): g_1(\mathbf {x},\mathbf {z}) \ge 0 \wedge \cdots \wedge g_n(\mathbf {x},\mathbf {z}) \! \ge 0$$,

$\mathbf {x}\in \mathbb {R}^r$, $\mathbf {y}\in \mathbb {R}^s$, $\mathbf {z}\in \mathbb {R}^t$ are variable vectors, $r,s,t \in \mathbb {N}$, and $f_1,\ldots ,f_m,g_1,\ldots ,g_n$ are polynomials. In addition, $\mathcal {M}_{\mathbf {x},\mathbf {y}}\{ f_1(\mathbf {x},\mathbf {y}),$ $\ldots , f_m(\mathbf {x},\mathbf {y}) \}$ and $\mathcal {M}_{\mathbf {x},\mathbf {z}}\{ g_1(\mathbf {x},\mathbf {z})$, $\ldots$, $g_n(\mathbf {x},\mathbf {z}) \}$ are two Archimedean quadratic modules. Here we allow uncommon variables, that are not allowed in [7], and drop the constraint that polynomials must be concave and quadratic, which is assumed in [10]. The Archimedean condition amounts to that all the variables are bounded, which is reasonable in program verification, as only bounded numbers can be represented in computer in practice. We first prove that there exists a polynomial $h(\mathbf {x})$ such that $h(\mathbf {x})=0$ separates the state space of $\mathbf {x}$ defined by $\phi (\mathbf {x},\mathbf {y})$ from the one defined by $\psi (\mathbf {x},\mathbf {z})$ theoretically, and then propose an algorithm to compute such $h(\mathbf {x})$ based on $\mathrm{SDP}$. Furthermore, we propose a verification approach to assure the validity of the synthesized interpolant and consequently avoid the unsoundness caused by numerical error in $\mathrm{SDP}$ solving. Finally, we also discuss how to extend our results to general semi-algebraic constraints.

Another contribution of this paper is that as an application, we illustrate how to apply our approach to invariant generation in program verification by revising Lin *et al.*’s framework proposed in [22] for invariant generation based on *weakest precondition*, *strongest postcondition* and *interpolation* by allowing to generate nonlinear invariants.

The paper is organized as follows. Some preliminaries and the problem of interest are introduced in Sect. 2. Section 3 shows the existence of an interpolant for two mutually contradictory polynomial formulas only containing conjunction, and Sect. 4 presents SDP-based methods to compute it. In Sect. 5, we discuss how to avoid unsoundness caused by numerical error in SDP. Section 6 extends our approach to general polynomial formulas. Section 7 demonstrates how to apply our approach to invariant generation in program verification. We conclude this paper in Sect. 8.

## Preliminaries

In this section, we first give a brief introduction on some notions used throughout this paper and then describe the problem of interest.

### Quadratic Module

$\mathbb {N}$, $\mathbb {Q}$ and $\mathbb {R}$ are the sets of integers, rational numbers and real numbers, respectively. $\mathbb {Q}[\mathbf {x}]$ and $\mathbb {R}[\mathbf {x}]$ denotes the polynomial ring over rational numbers and real numbers in $r\ge 1$ indeterminates $\mathbf {x}:(x_1,\ldots ,x_r)$. We use $\mathbb {R}[\mathbf {x}]^2:=\{p^2\mid p\in \mathbb {R}[\mathbf {x}]\}$ for the set of squares and $\sum \mathbb {R}[\mathbf {x}]^2$ for the set of sums of squares of polynomials in $\mathbf {x}$. Vectors are denoted by boldface letters. $\bot$ and $\top$ stand for **false** and **true**, respectively.

#### Definition 1

**(Quadratic Module** [24]**).** A subset $\mathcal {M}$ of $\mathbb {R}[\mathbf {x}]$ is called a *quadratic module* if it contains 1 and is closed under addition and multiplication with squares, i.e., $1\in \mathcal {M}, \mathcal {M} + \mathcal {M} \subseteq \mathcal {M}$, and $p^2 \mathcal {M} \subseteq \mathcal {M}$ for all $p \in \mathbb {R}[\mathbf {x}]$.

Let $\overline{p}:=\{p_1,\ldots ,p_s\}$ be a finite subset of $\mathbb {R}[\mathbf {x}]$, the quadratic module $\mathcal {M}_{\mathbf {x}}( \overline{p})$ or simply $\mathcal {M}(\overline{p})$ generated by $\overline{p}$ (i.e. the smallest quadratic module containing all $p_i$s) is $\mathcal {M}_{\mathbf {x}}(\overline{p})=\{\sum _{i=0}^s \delta _i p_i\mid \delta _i \in \sum \mathbb {R}[\mathbf {x}]^2\}$, where $p_0=1$.

Archimedean condition plays a key role in the study of polynomial optimization.

#### Definition 2

**(Archimedean).** Let $\mathcal {M}$ be a quadratic module of $\mathbb {R}[\mathbf {x}]$. $\mathcal {M}$ is said to be *Archimedean* if there exists some $a>0$ such that $a-\sum _{i=1}^{r} x_i^2 \in \mathcal {M}$.

### Problem Description

Craig showed that given two formulas $\phi$ and $\psi$ in a first-order theory $\mathcal {T}$, if $\phi \models \psi$, then there always exists an *interpolant* *I* over the common symbols of $\phi$ and $\psi$ s.t. $\phi \models I$ and $I \models \psi$. In the verification literature, this terminology has been abused following [26], where a *reverse interpolant* (coined by Kovács and Voronkov in [19]) *I* over the common symbols of $\phi$ and $\psi$ is defined by

#### Definition 3

**(Interpolant).** Given two formulas $\phi$ and $\psi$ in a theory $\mathcal {T}$ s.t. $\phi \wedge \psi \models _{\mathcal {T}} \bot$, a formula *I* is an *interpolant* of $\phi$ and $\psi$ if (i) $\phi \models _{\mathcal {T}} I$; (ii) $I \wedge \psi \models \bot$; and (iii) *I* only contains common symbols and free variables shared by $\phi$ and $\psi$.

#### Definition 4

A basic semi-algebraic set $\{\mathbf {x}\in \mathbb {R}^n\mid \bigwedge _{i=1}^s p_i(\mathbf {x})\ge 0\}$ is called a set of the *Archimedean form* if $\mathcal {M}_{\mathbf {x}}\{p_1(\mathbf {x}),\ldots ,p_s(\mathbf {x})\}$ is Archimedean, where $p_i(\mathbf {x})\in \mathbb {R}[\mathbf {x}]$, $i=1,\ldots ,s$.

The interpolant synthesis problem of interest in this paper is given in Problem 1.

#### Problem 1

Let $\phi (\mathbf {x},\mathbf {y})$ and $\psi (\mathbf {x},\mathbf {z})$ be two polynomial formulas of the form

$$\begin{aligned} \phi (\mathbf {x},\mathbf {y}) : f_1(\mathbf {x},\mathbf {y}) \ge 0 \wedge \cdots \wedge f_m(\mathbf {x},\mathbf {y})\ge 0, \\ \psi (\mathbf {x},\mathbf {z}) : g_1(\mathbf {x},\mathbf {z}) \ge 0 \wedge \cdots \wedge g_n(\mathbf {x},\mathbf {z})\ge 0, \end{aligned}$$

where, $\mathbf {x}\in \mathbb {R}^r$, $\mathbf {y}\in \mathbb {R}^s$, $\mathbf {z}\in \mathbb {R}^t$ are variable vectors, $r,s,t \in \mathbb {N}$, and $f_1,\ldots ,f_m,g_1$, ..., $g_n$ are polynomials in the corresponding variables. Suppose $\phi \wedge \psi \models \bot$, and $\{(\mathbf {x},\mathbf {y})\mid \phi (\mathbf {x},\mathbf {y})\}$ and $\{(\mathbf {x},\mathbf {z})\mid \psi (\mathbf {x},\mathbf {z})\}$ are semi-algebraic sets of the Archimedean form. Find a polynomial $h(\mathbf {x})$ such that $h(\mathbf {x})>0$ is an interpolant for $\phi$ and $\psi$.

## Existence of Interpolants

The basic idea and steps of proving the existence of interpolants are as follows: Because an interpolant of $\phi$ and $\psi$ contains only the common symbols in $\phi$ and $\psi$, it is natural to consider the projections of the sets defined by $\phi$ and $\psi$ on $\mathbf {x}$, i.e. $P_{\mathbf {x}}(\phi (\mathbf {x},\mathbf {y})) \hat{=} \{ \mathbf {x}\mid \exists \mathbf {y}. \, \phi (\mathbf {x},\mathbf {y})\}$ and $P_{\mathbf {x}}(\psi (\mathbf {x},\mathbf {z})) \hat{=} \{ \mathbf {x}\mid \exists \mathbf {z}.\, \psi (\mathbf {x},\mathbf {z})\}$, which are obviously disjoint. We therefore prove that, if $h(\mathbf {x})=0$ separates $P_{\mathbf {x}}(\phi (\mathbf {x},\mathbf {y}))$ and $P_{\mathbf {x}}(\psi (\mathbf {x},\mathbf {z}))$, then $h(\mathbf {x})$ solves Problem 1 (see Proposition 1). Thus, we only need to prove the existence of such $h(\mathbf {x})$ through the following steps: First, we prove that $P_{\mathbf {x}}(\phi (\mathbf {x},\mathbf {y}))$ and $P_{\mathbf {x}}(\psi (\mathbf {x},\mathbf {z}))$ are compact semi-algebraic sets which are unions of finitely many basic closed semi-algebraic sets (see Lemma 1). Second, using Putinar’s Positivstellensatz, we prove that, for two disjoint basic closed semi-algebraic sets $S_1$ and $S_2$ of the Archimedean form, there exists a polynomial $h_1(\mathbf {x})$ such that $h_1(\mathbf {x})=0$ separates $S_1$ and $S_2$ (see Lemma 2). This result is then extended to the case that $S_2$ is a finite union of basic closed semi-algebraic sets (see Lemma 3). Finally, by generalizing Lemma 3 to the case that two compact semi-algebraic sets both are unions of finitely many basic closed semi-algebraic sets and together with Proposition 1, we prove the existence of interpolant in Theorem 2 and Corollary 1.

### Proposition 1

If $h(\mathbf {x})\in \mathbb {R}[\mathbf {x}]$ satisfies the following constraints

1 $$\begin{aligned} \forall \mathbf {x}\in P_{\mathbf {x}}(\phi (\mathbf {x},\mathbf {y})). h(\mathbf {x}) > 0 ~~ \text{ and } ~~ \forall \mathbf {x}\in P_{\mathbf {x}}(\psi (\mathbf {x},\mathbf {z})). h(\mathbf {x}) < 0, \end{aligned}$$

then $h(\mathbf {x})> 0$ is an interpolant for $\phi (\mathbf {x},\mathbf {y})$ and $\psi (\mathbf {x},\mathbf {z})$, where $\phi (\mathbf {x},\mathbf {y})$ and $\psi (\mathbf {x},\mathbf {z})$ are defined as in Problem 1.

### Proof

According to Definition 3, it is enough to prove that $\phi (\mathbf {x},\mathbf {y}) \models h(\mathbf {x})>0$ and $\psi (\mathbf {x},\mathbf {z}) \models h(\mathbf {x}) \le 0$.

Since any $(\mathbf {x}_0,\mathbf {y}_0)$ satisfying $\phi (\mathbf {x},\mathbf {y})$ must imply $\mathbf {x}_0 \in P_{\mathbf {x}}(\phi (\mathbf {x},\mathbf {y}))$, it follows that $h(\mathbf {x}_0)>0$ from (1) and $\phi (\mathbf {x},\mathbf {y}) \models h(\mathbf {x})>0$. Similarly, we can prove $\psi (\mathbf {x},\mathbf {z}) \models h(\mathbf {x})<0$, implying that $\psi (\mathbf {x},\mathbf {z}) \models h(\mathbf {x}) \le 0$. Therefore, $h(\mathbf {x})> 0$ is an interpolant for $\phi (\mathbf {x},\mathbf {y})$ and $\psi (\mathbf {x},\mathbf {z})$.    $\square$

In order to synthesize such $h(\mathbf {x})$ in Proposition 1, we first dig deeper into the two sets $P_{\mathbf {x}}(\phi (\mathbf {x},\mathbf {y}))$ and $P_{\mathbf {x}}(\psi (\mathbf {x},\mathbf {z}))$. As shown later, i.e. in Lemma 1, we will find that these two sets are compact semi-algebraic sets of the form $\{\mathbf {x}\mid \bigvee _{i=1}^{c} \bigwedge _{j=1}^{J_i} \alpha _{i,j}(\mathbf {x}) \ge 0\}$. Before this lemma, we introduce Finiteness theorem pertinent to a *basic closed semi-algebraic subset* of $\mathbb {R}^n$, which will be used in the proof of Lemma 1, where a basic closed semi-algebraic subset of $\mathbb {R}^n$ is a set of the form $\{\mathbf {x}\in \mathbb {R}^n\mid \alpha _1(\mathbf {x})\ge 0,\ldots ,\alpha _k(\mathbf {x})\ge 0\}$ with $\alpha _1,\ldots ,\alpha _k\in \mathbb {R}[\mathbf {x}]$.

### Theorem 1

**(Finiteness Theorem, Theorem 2.7.2 in** [3]**).** Let $A\subset \mathbb {R}^n$ be a closed semi-algebraic set. Then *A* is a finite union of basic closed semi-algebraic sets.

### Lemma 1

The set $P_{\mathbf {x}}(\phi (\mathbf {x},\mathbf {y}))$ is compact semi-algebraic set of the following form

$$\begin{aligned} P_{\mathbf {x}}(\phi (\mathbf {x},\mathbf {y})):=\{\mathbf {x}\mid \bigvee _{i=1}^{c} \bigwedge _{j=1}^{J_i} \alpha _{i,j}(\mathbf {x}) \ge 0\}, \end{aligned}$$

where $\alpha _{i,j}(\mathbf {x})\in \mathbb {R}[\mathbf {x}]$, $i=1,\ldots ,c$, $j=1,\ldots ,J_i$. The same claim applies to the set $P_{\mathbf {x}}(\psi (\mathbf {x},\mathbf {z}))$ as well.

### Proof

For the sake of simplicity, we denote $\{(\mathbf {x},\mathbf {y})\mid \phi (\mathbf {x},\mathbf {y})\}$ and $P_{\mathbf {x}}(\phi (\mathbf {x},\mathbf {y}))$ by *S* and $\pi (S)$, respectively.

Because *S* is a compact set and $\pi$ is a continuous map that maps compact set to compact set, $\pi (S)$, which is the image of a compact set under a continuous map, is compact. Moreover, as *S* is a semi-algebraic set and the projection of a semi-algebraic set is also a semi-algebraic set by Tarski-Seidenberg theorem [2], this implies that $\pi (S)$ is a semi-algebraic set. Thus, $\pi (S)$ is a compact semi-algebraic set.

Since $\pi (S)$ is a compact semi-algebraic set, and also a closed semi-algebraic set, we have that $\pi (\mathrm {S})$ is a finite union of basic closed semi-algebraic sets from Theorem 1. Hence, there exist a series of polynomials $\alpha _{1,1}(\mathbf {x}), \ldots , \alpha _{1,J_1}(\mathbf {x}), \ldots , \alpha _{c,1}(\mathbf {x}), \ldots , \alpha _{c,J_c}(\mathbf {x})$ such that $\pi (\mathrm {S})=\bigcup _{i=1}^{c}\{\mathbf {x}\mid \bigwedge _{j=1}^{J_i} \alpha _{i,j}(\mathbf {x}) \ge 0\} =\{\mathbf {x}\mid \bigvee _{i=1}^{c}\bigwedge _{j=1}^{J_i} \alpha _{i,j}(\mathbf {x}) \ge 0\}$. This concludes this lemma.    $\square$

After knowing the structure of $P_{\mathbf {x}}(\phi (\mathbf {x},\mathbf {y}))$ and $P_{\mathbf {x}}(\psi (\mathbf {x},\mathbf {z}))$ being a union of some basic semialgebraic sets as illustrated in Lemma 1, we next prove the existence of $h(\mathbf {x})\in \mathbb {R}[\mathbf {x}]$ satisfying (1), as formally stated in Theorem 2.

### Theorem 2

Suppose that $\phi (\mathbf {x},\mathbf {y})$ and $\psi (\mathbf {x},\mathbf {z})$ are defined as in Problem 1, then there exists a polynomial $h(\mathbf {x})$ satisfying (1).

As pointed out by an anonymous reviewer that Theorem 2 can be obtained by some properties of the ring of Nash functions proved in [29]. In what follows, we give a simpler and more intuitive proof. To the end, it requires some preliminaries first. The main tool in our proof is Putinar’s Positivstellensatz, as formulated in Theorem 3.

### Theorem 3

**(Putinar’s Positivstellensatz** [31]**).** Let $p_1,\ldots ,p_k \in \mathbb {R}[\mathbf {x}]$ and $S_1=\{\mathbf {x}\mid p_1(\mathbf {x})\ge 0, \ldots , p_k(\mathbf {x}) \ge 0 \}$. Assume that the quadratic module $\mathcal {M}(p_1,\ldots ,p_k)$ is Archimedean. For $q\in \mathbb {R}[\mathbf {x}]$, if $q>0$ on $S_1$ then $q\in \mathcal {M}(p_1,\ldots ,p_k)$.

With Putinar’s Positivstellensatz we can draw a conclusion that there exists a polynomial such that its zero level set^3^ separates two compact semi-algebraic sets of the Archimedean form, as claimed in Lemmas 2 and 3.

### Lemma 2

Let $S_1=\{\mathbf {x}\mid p_1(\mathbf {x})\ge 0, \ldots , p_J(\mathbf {x})\ge 0\},$ $S_2=\{\mathbf {x}\mid q_1(\mathbf {x})\ge 0, \ldots , q_K(\mathbf {x})\ge 0\}$ be semi-algebraic sets of the Archimedean form and $S_1 \cap S_2 = \emptyset$, then there exists a polynomial $h_1(\mathbf {x})$ such that

2 $$\begin{aligned} \forall \mathbf {x}\in S_1.~ h_1(\mathbf {x})>0, \quad \forall \mathbf {x}\in S_2.~ h_1(\mathbf {x})<0. \end{aligned}$$

### Proof

Since $S_1 \cap S_2 = \emptyset$, it follows

$$p_2\ge 0\wedge \cdots \wedge p_J \ge 0 \wedge q_1\ge 0\wedge \cdots \wedge q_K \ge 0 \models -p_1> 0.$$

Let $S_3= \{\mathbf {x}\mid p_2\ge 0\wedge \cdots \wedge p_J \ge 0 \wedge q_1\ge 0\wedge \cdots \wedge q_K \ge 0\}$, then $-p_1>0$ on $S_3$. Since $S_1$ and $S_2$ are semi-algebraic sets of the Archimedean form, it follows $\mathcal {M}_{\mathbf {x}}(p_2(\mathbf {x}),\ldots ,p_J(\mathbf {x}),q_1(\mathbf {x}), \ldots , q_K(\mathbf {x}))$ is also Archimedean. Hence, $S_3$ is compact. From $-p_1>0$ on $S_3$, we further have that there exists some $u_1 \in \sum \mathbb {R}[\mathbf {x}]^2$ such that $-u_1p_1-1>0$ on $S_3$. Using Theorem 3, we have that

$$-u_1p_1-1 \in \mathcal {M}_{\mathbf {x}}(p_2(\mathbf {x}),\ldots ,p_J(\mathbf {x}),q_1(\mathbf {x}), \ldots , q_K(\mathbf {x})),$$

implying that there exists a set of sums of squares polynomials $u_2,\ldots ,u_J$ and $v_0$,$v_1$, ..., $v_K \in \mathbb {R}[\mathbf {x}]$, such that

$$-u_1p_1-1\equiv u_2p_2+\cdots +u_Jp_J+v_0+v_1q_1+\cdots +v_Kq_K.$$

Let $h_1=\frac{1}{2}+u_1p_1+\cdots +u_Jp_J$, i.e., $-h_1=\frac{1}{2}+v_0+v_1q_1+\cdots +v_Kq_K$. It is easy to check that (2) holds.    $\square$

Lemma 3 generalizes the result of Lemma 2 to more general compact semi-algebraic sets of the Archimedean form, which is the union of multiple basic semi-algebraic sets.

### Lemma 3

Assume $S_0=\{\mathbf {x}\mid p_1(\mathbf {x})\ge 0, \ldots , p_J(\mathbf {x})\ge 0\}$ and $S_i=\{\mathbf {x}\mid q_{i,1}(\mathbf {x})\ge 0, \ldots , q_{i,K_i}(\mathbf {x})\ge 0\}$, $i=1,\ldots ,b$, are semi-algebraic sets of the Archimedean form, and $S_0 \cap \bigcup _{i=1}^{b}S_i = \emptyset$, then there exists a polynomial $h_0(\mathbf {x})$ such that

3 $$\begin{aligned} \forall \mathbf {x}\in S_0. ~h_0(\mathbf {x})>0, \quad \forall \mathbf {x}\in \bigcup _{i=1}^{b}S_i. ~ h_0(\mathbf {x})<0. \end{aligned}$$

In order to prove this lemma, we prove the following lemma first.

### Lemma 4

Let $c,d \in \mathbb {R}$ with $0<c<d$ and $U_0=[c,d]^r$. There exists a polynomial $\hat{h}(\mathbf {x})$ such that

4 $$\begin{aligned} \mathbf {x}\in U_0 \models \hat{h}(\mathbf {x})>0 \models \bigwedge _{i=1}^{r} x_i > 0, \end{aligned}$$

where $$\mathbf {x}=(x_1,\ldots ,x_r)$$.

### Proof

We show that there exists $k \in \mathbb {N}$ such that $\hat{h}(\mathbf {x})=(\frac{d}{2})^{2k}-(x_1-\frac{c+d}{2})^{2k}-\cdots -(x_r-\frac{c+d}{2})^{2k}$ satisfies (4). It is evident that $\hat{h}(\mathbf {x})>0 \models \bigwedge _{i=1}^r x_i>0$ holds. In the following we just need to verify that $\bigwedge _{i=1}^r c\le x_i \le d \models \hat{h}(\mathbf {x})>0$ holds. Since $c\le x_i \le d$, we have $(x_i-\frac{c+d}{2})^{2k} \le (\frac{d-c}{2})^{2k}$ and $(\frac{d}{2})^{2k}-\sum _{i=1}^{r}(x_i-\frac{c+d}{2})^{2k}\ge (\frac{d}{2})^{2k}-r(\frac{d-c}{2})^{2k}.$ Obviously, if an integer *k* satisfies $(\frac{d}{d-c})^{2k}> r$, then $(\frac{d}{2})^{2k}-\sum _{i=1}^{r}(x_i-\frac{c+d}{2})^{2k}>0$. The existence of such *k* satisfying $(\frac{d}{d-c})^{2k}> r$ is assured by $\frac{d}{d-c}> 1$.    $\square$

Now we give a proof for Lemma 3 as follows.

### Proof

*(of Lemma* 3*).* For any *i* with $1\le i\le b$, according to Lemma 2, there exists a polynomial $h_i\in \mathbb {R}[\mathbf {x}]$, satisfying $\forall \mathbf {x}\in S_0.~ h_i(\mathbf {x})>0$ and $\forall \mathbf {x}\in S_i. ~ h_i(\mathbf {x})<0$.

Next, we construct $h_0(\mathbf {x})\in \mathbb {R}[\mathbf {x}]$ from $h_1(\mathbf {x}), \ldots , h_b(\mathbf {x})$. Since $S_0$ is a semi-algebraic set of the Archimedean form, $S_0$ is compact and thus $h_i(\mathbf {x})$ has minimum value and maximum value on $S_0$, denoted by $c_i$ and $d_i$ respectively. Let $c=\min (c_1, \ldots , c_b)$ and $d=\max (d_1, \ldots , d_b)$. Clearly, $0<c<d$.

From Lemma 4 there must exist a polynomial $\hat{h}(w_1,\ldots ,w_b)$ such that

5 $$\begin{aligned} \bigwedge _{i=1}^b c\le w_i \le d \models \hat{h}(w_1,\ldots ,w_b)>0, \end{aligned}$$

6 $$\begin{aligned} \hat{h}(w_1,\ldots ,w_b)>0 \models \bigwedge _{i=1}^b w_i>0. \end{aligned}$$

Let $h'_0(\mathbf {x})=\hat{h}(h_1(\mathbf {x}), \ldots ,h_b(\mathbf {x}))$. Obviously, $h'_0(\mathbf {x})\in \mathbb {R}[\mathbf {x}]$. We next prove that $h'_0(\mathbf {x})$ satisfies (3) in Lemma 3.

For all $\mathbf {x}_0\in S_0$, $c \le h_i(\mathbf {x}_0) \le d$, $i=1,\ldots ,b$, $h'_0(\mathbf {x}_0)=\hat{h}(h_1(\mathbf {x}_0), \ldots ,h_b(\mathbf {x}_0)) >0$ by (5). Therefore, the first constraint in (3), i.e. $\forall \mathbf {x}_0\in S_0. h_0(\mathbf {x}_0) >0$, holds.

For any $\mathbf {x}_0\in \bigcup _{i=1}^{b} S_i$, there must exist some *i* such that $\mathbf {x}_0 \in S_i$, implying that $h_i(\mathbf {x}_0)<0$. By (6) we have $h'_0(\mathbf {x}_0)=\hat{h}(h_1(\mathbf {x}_0), \ldots ,h_b(\mathbf {x}_0)) \le 0$.

Thus, we obtain the conclusion that there exists a polynomial $h'_0(\mathbf {x})$ such that $\forall \mathbf {x}\in S_0.~h'_0(\mathbf {x})>0$, and $\forall \mathbf {x}\in \bigcup _{i=1}^{b}S_i.~ h'_0(\mathbf {x})\le 0$. Also, since $S_0$ is a compact set, and $h'_0(\mathbf {x})>0$ on $S_0$, there must exist some positive number $\epsilon >0$ such that $h'_0(\mathbf {x})-\epsilon >0$ over $S_0$. Then $h'_0(\mathbf {x})-\epsilon < 0$ on $\bigcup _{i=1}^{b}S_i$. Therefore, setting $h_0(\mathbf {x}):=h'_0(\mathbf {x})-\epsilon$, Lemma 3 is proved.    $\square$

In Lemma 3 we proved that there exists a polynomial $h(\mathbf {x}) \in \mathbb {R}[\mathbf {x}]$ such that its zero level set is a barrier between two semi-algebraic sets of the Archimedean form, of which one set is a union of finitely many basic semi-algebraic sets. In the following we will give a formal proof of Theorem 2, which is a generalization of Lemma 3.

### Proof

*(of Theorem* 2*).* According to Lemma 1 we have that $P_{\mathbf {x}}(\phi (\mathbf {x},\mathbf {y}))$ and $P_{\mathbf {x}}(\psi (\mathbf {x},\mathbf {z}))$ are compact sets, and there respectively exists a set of polynomials $p_{i,j}(\mathbf {x})\in \mathbb {R}[\mathbf {x}]$, $i=1,\ldots ,a$, $j=1,\ldots ,J_i$, and $q_{l,k}(\mathbf {x})\in \mathbb {R}[\mathbf {x}]$, $l=1,\ldots ,b$, $k=1,\ldots ,K_i$, such that

$$\begin{aligned} P_{\mathbf {x}}(\phi (\mathbf {x},\mathbf {y}))=\{\mathbf {x}\mid \bigvee _{i=1}^{a} \bigwedge _{j=1}^{J_i} p_{i,j}(\mathbf {x}) \ge 0\}, \quad P_{\mathbf {x}}(\psi (\mathbf {x},\mathbf {z}))=\{\mathbf {x}\mid \bigvee _{l=1}^{b} \bigwedge _{k=1}^{K_l} q_{l,k}(\mathbf {x}) \ge 0\}. \end{aligned}$$

Since $P_{\mathbf {x}}(\phi (\mathbf {x},\mathbf {y}))$ and $P_{\mathbf {x}}(\psi (\mathbf {x},\mathbf {z}))$ are compact sets, there exists a positive $N\in \mathbb {R}$ such that $f=N-\sum _{i=1}^rx_i^2\ge 0$ over $P_{\mathbf {x}}(\phi (\mathbf {x},\mathbf {y}))$ and $P_{\mathbf {x}}(\psi (\mathbf {x},\mathbf {z}))$. For each $i=1,\ldots ,a$ and each $l=1,\ldots ,b$, set $p_{i,0}=q_{l,0}=f$. Denote $\{\mathbf {x}\mid \bigvee _{i=1}^{a} \bigwedge _{j=0}^{J_i} p_{i,j}(\mathbf {x}) \ge 0\}=\bigcup _{i=1}^a\{\mathbf {x}\mid \bigwedge _{j=0}^{J_i} p_{i,j}(\mathbf {x}) \ge 0\}$ by $P_1$ and $\{\mathbf {x}\mid \bigvee _{l=1}^{b} \bigwedge _{k=0}^{K_l} q_{l,k}(\mathbf {x}) \ge 0\}=\bigcup _{l=1}^b \{\mathbf {x}\mid \bigwedge _{k=0}^{K_l} q_{l,k}(\mathbf {x}) \ge 0\}$ by $P_2$. It is easy to see that $P_1=P_{\mathbf {x}}(\phi (\mathbf {x},\mathbf {y})$, $P_2=P_{\mathbf {x}}(\psi (\mathbf {x},\mathbf {z}))$.

Since $\phi \wedge \psi \models \bot$, there does not exist $(\mathbf {x},\mathbf {y},\mathbf {z})\in \mathbb {R}^{r+s+t}$ that satisfies $\phi \wedge \psi$, implying that $P_{\mathbf {x}}(\phi (\mathbf {x},\mathbf {y})) \cap P_{\mathbf {x}}(\psi (\mathbf {x},\mathbf {z}))=\emptyset$ and thus $P_1\,\cap \, P_2=\emptyset$. Also, since $\{ \mathbf {x}\mid \bigwedge _{j=0}^{J_{i_1}} p_{i_1,j}(\mathbf {x}) \ge 0\}\subseteq P_1$, for each $i_1=1,\ldots ,a$, $\{\mathbf {x}\mid \bigwedge _{j=0}^{J_{i_1}} p_{i_1,j}(\mathbf {x}) \ge 0\} \cap P_2=\emptyset$ holds. By Lemma 3 there exists $h_{i_1}(\mathbf {x})\in \mathbb {R}[\mathbf {x}]$ such that

$$\begin{aligned} \forall \mathbf {x}\in \{ \mathbf {x}\mid \bigwedge _{j=0}^{J_{i_1}} p_{i_1,j}(\mathbf {x}) \ge 0\}. h_{i_1}(\mathbf {x})>0, \quad \forall \mathbf {x}\in P_2. h_{i_1}(\mathbf {x})<0. \end{aligned}$$

Let $S'=\{\mathbf {x}\mid -h_1(\mathbf {x})\ge 0, \ldots , -h_a(\mathbf {x})\ge 0, N-\sum _{i=1}^rx_i^2\ge 0\}$. Obviously, $S'$ is a semialgebraic set of the Archimedean form, $P_2\subset S'$ and $P_1 \cap S'=\emptyset .$ Therefore, according to Lemma 2, there exists a polynomial $\overline{h}(\mathbf {x})\in \mathbb {R}[\mathbf {x}]$ such that $\forall \mathbf {x}\in S'.~ \overline{h}(\mathbf {x}) >0$ and $\forall \mathbf {x}\in P_1.~\overline{h}(\mathbf {x})<0$. Let $h(\mathbf {x})=-\overline{h}(\mathbf {x})$, then we have $\forall \mathbf {x}\in P_1.~h(\mathbf {x})>0$ and $\forall \mathbf {x}\in P_2.~h(\mathbf {x})<0$, implying that $\forall \mathbf {x}\in P_{\mathbf {x}}(\phi (\mathbf {x},\mathbf {y})). h(\mathbf {x}) > 0$ and $\forall \mathbf {x}\in P_{\mathbf {x}}(\psi (\mathbf {x},\mathbf {z})). h(\mathbf {x}) < 0$. Thus, this completes the proof of Theorem 2.    $\square$

Consequently, we immediately have the following conclusion.

### Corollary 1

Let $\phi (\mathbf {x},\mathbf {y})$ and $\psi (\mathbf {x},\mathbf {z})$ be defined as in Problem 1. There must exist a polynomial $h(\mathbf {x})\in \mathbb {R}[\mathbf {x}]$ such that $h(\mathbf {x})>0$ is an interpolant for $\phi$ and $\psi$.

Actually, since $P_{\mathbf {x}}(\phi (\mathbf {x},\mathbf {y}))$ and $P_{\mathbf {x}}(\psi (\mathbf {x},\mathbf {z}))$ both are compact set by Lemma 1, and $h(\mathbf {x})>0$ on $P_{\mathbf {x}}(\phi (\mathbf {x},\mathbf {y}))$ and $h(\mathbf {x})<0$ on $P_{\mathbf {x}}(\psi (\mathbf {x},\mathbf {z}))$, we can obtain $h'(\mathbf {x})$ by giving a small perturbation to the coefficients of $h(\mathbf {x})$ such that $h'(\mathbf {x})$ has the property of $h(\mathbf {x})$. Hence, there should exist a $h(\mathbf {x}) \in \mathbb {Q}[\mathbf {x}]$ such that $h(\mathbf {x})>0$ is an interpolant for $\phi$ and $\psi$, intuitively.

### Theorem 4

Let $\phi (\mathbf {x},\mathbf {y})$ and $\psi (\mathbf {x},\mathbf {z})$ be defined as in Problem 1. There must exist a polynomial $h(\mathbf {x})\in \mathbb {Q}[\mathbf {x}]$ such that $h(\mathbf {x})>0$ is an interpolant for $\phi$ and $\psi$.

### Proof

We just need to prove there exists a polynomial $h(\mathbf {x})\in \mathbb {Q}[\mathbf {x}]$ satisfying (1).

By Theorem 2, there exists a polynomial $h'(\mathbf {x})\in \mathbb {R}[\mathbf {x}]$ satisfying (1). Since $P_{\mathbf {x}}(\phi (\mathbf {x},\mathbf {y}))$ and $P_{\mathbf {x}}(\psi (\mathbf {x},\mathbf {z}))$ are compact sets, $h'(\mathbf {x})>0$ on $P_{\mathbf {x}}(\phi (\mathbf {x},\mathbf {y}))$ and $h'(\mathbf {x})<0$ on $P_{\mathbf {x}}(\psi (\mathbf {x},\mathbf {z}))$, there exist $\eta _1>0$ and $\eta _2>0$ such that

$$\begin{aligned} \forall \mathbf {x}\in P_{\mathbf {x}}(\phi (\mathbf {x},\mathbf {y})). h'(\mathbf {x})-\eta _1 \ge 0,~ \forall \mathbf {x}\in P_{\mathbf {x}}(\psi (\mathbf {x},\mathbf {z})). h'(\mathbf {x})+\eta _2 \le 0. \end{aligned}$$

Let $\eta =\min (\frac{\eta _1}{2},\frac{\eta _2}{2})$. Suppose $h'(\mathbf {x})\in \mathbb {R}[\mathbf {x}]$ has the form $h'(\mathbf {x})=\sum _{\alpha \in \varOmega } c_{\alpha }\mathbf {x}^{\alpha }$, where $\alpha \in \mathbb {N}^r$, $\varOmega \subset \mathbb {N}^r$ is a finite set of indices, *r* is the dimension of $\mathbf {x}$, $\mathbf {x}^{\alpha }$ is the monomial $\mathbf {x}_1^{\alpha _1}\cdots \mathbf {x}_r^{\alpha _r}$, and $0\ne c_{\alpha }\in \mathbb {R}$ is the coefficient of monomial $\mathbf {x}^{\alpha }$. Let $N=|\varOmega |$ be the cardinality of $\varOmega$. Since $P_{\mathbf {x}}(\phi (\mathbf {x},\mathbf {y}))$ and $P_{\mathbf {x}}(\psi (\mathbf {x},\mathbf {z}))$ are compact sets, for any $\alpha \in \varOmega$, there exists $M_{\alpha }>0$ such that $M_{\alpha }=\max \{|\mathbf {x}^{\alpha }| \mid \mathbf {x}\in P_{\mathbf {x}}(\phi (\mathbf {x},\mathbf {y})) \cup P_{\mathbf {x}}(\psi (\mathbf {x},\mathbf {z}))\}$. Then for any fixed polynomial $\hat{h}(\mathbf {x})=\sum _{\alpha \in \varOmega } d_{\alpha }\mathbf {x}^{\alpha }$, with $d_{\alpha }\in [c_{\alpha }-\frac{\eta }{NM_{\alpha }},c_{\alpha }+\frac{\eta }{NM_{\alpha }}]$, and any $\mathbf {x}\in P_{\mathbf {x}}(\phi (\mathbf {x},\mathbf {y})) \cup P_{\mathbf {x}}(\psi (\mathbf {x},\mathbf {z}))$, we have

$$\begin{aligned} |\hat{h}(\mathbf {x})-h'(\mathbf {x})|&=|\sum _{\alpha \in \varOmega } (d_{\alpha }-c_{\alpha })\mathbf {x}^{\alpha }| \le \sum _{\alpha \in \varOmega } |(d_{\alpha }-c_{\alpha })|\cdot |\mathbf {x}^{\alpha }| \le \sum _{\alpha \in \varOmega }\frac{\eta }{NM_{\alpha }} \cdot M_{\alpha }=\eta . \end{aligned}$$

Since $$\eta = \min (\frac{\eta _1}{2},\frac{\eta _2}{2})$$, hence

7 $$\begin{aligned} \forall \mathbf {x}\in P_{\mathbf {x}}(\phi (\mathbf {x},\mathbf {y})). \hat{h}(\mathbf {x})\ge \frac{\eta _1}{2} > 0,\quad \forall \mathbf {x}\in P_{\mathbf {x}}(\psi (\mathbf {x},\mathbf {z})). \hat{h}(\mathbf {x}) \le -\frac{\eta _2}{2} < 0. \end{aligned}$$

Since for any $d_\alpha \in [c_{\alpha }-\frac{\eta }{NM_{\alpha }},c_{\alpha }+\frac{\eta }{NM_{\alpha }}]$ (7) holds, there must exist some rational number $r_{\alpha }\in \mathbb {Q}$ in $[c_{\alpha }-\frac{\eta }{NM_{\alpha }},c_{\alpha }+\frac{\eta }{NM_{\alpha }}]$ satisfying (7) because of the density of rational numbers. Thus, let $h(\mathbf {x})=\sum _{\alpha \in \varOmega } r_{\alpha }\mathbf {x}^{\alpha }$. Clearly, it follows that $h(\mathbf {x})\in \mathbb {Q}[\mathbf {x}]$ and (1) holds.    $\square$

So, the existence of $h(\mathbf {x})\in \mathbb {Q}[\mathbf {x}]$ is guaranteed. Moreover, from the proof of Theorem 4, we know that a small perturbation of $h(\mathbf {x})$ is permitted, which is a good property for computing $h(\mathbf {x})$ in a numeric way. In the subsequent subsection, we recast the problem of finding such $h(\mathbf {x})$ as a semi-definite programming problem.

## SOS Formulation

Similar to [7], in this section, we discuss how to reduce the problem of finding $h(\mathbf {x})$ satisfying (1) to a sum of squares programming problem.

### Theorem 5

Let $\phi (\mathbf {x},\mathbf {y})$ and $\psi (\mathbf {x},\mathbf {z})$ be defined as in the Problem 1. Then there exist $m+n+2$ SOS (sum of squares) polynomials $u_i(\mathbf {x},\mathbf {y})~ (i=1,\ldots ,m+1),$ $v_j(\mathbf {x},\mathbf {z})~ (j=1,\ldots ,n+1)$ and a polynomial $h(\mathbf {x})$ such that

8 $$\begin{aligned} h-1=\sum _{i=1}^m u_if_i+u_{m+1}, \quad -h-1=\sum _{j=1}^nv_jg_j+ v_{n+1}, \end{aligned}$$

and $h(\mathbf {x})>0$ is an interpolant for $\phi (\mathbf {x},\mathbf {y})$ and $\psi (\mathbf {x},\mathbf {z})$.

### Proof

By Theorem 2 there exists a polynomial $\hat{h}(\mathbf {x})$ such that

$$\begin{aligned} \forall \mathbf {x}\in P_{\mathbf {x}}(\phi (\mathbf {x},\mathbf {y})). \hat{h}(\mathbf {x}) > 0, \quad \forall \mathbf {x}\in P_{\mathbf {x}}(\psi (\mathbf {x},\mathbf {z})). \hat{h}(\mathbf {x}) < 0. \end{aligned}$$

Set $S_1=\{(\mathbf {x},\mathbf {y})\mid f_1\ge 0, \ldots , f_m\ge 0\}$ and $S_2=\{(\mathbf {x},\mathbf {z})\mid g_1\ge 0, \ldots , g_n\ge 0\}$. Since $\hat{h}(\mathbf {x}) > 0$ on $S_1$, which is compact, there exist $\epsilon _1>0$ such that $\hat{h}(\mathbf {x})-\epsilon _1 > 0$ on $S_1$. Similarly, there exist $\epsilon _2>0$ such that $-\hat{h}(\mathbf {x})-\epsilon _2 > 0$ on $S_2$. Let $\epsilon =\min (\epsilon _1,\epsilon _2)$, and $h(\mathbf {x})=\frac{\hat{h}(\mathbf {x})}{\epsilon }$, then ${h}(\mathbf {x})-1 > 0$ on $S_1$ and $-{h}(\mathbf {x})-1 > 0$ on $S_2$. Since $\mathcal {M}_{\mathbf {x},\mathbf {y}}(f_1(\mathbf {x},\mathbf {y}), \ldots , f_m(\mathbf {x},\mathbf {y}))$ is Archimedean, from Theorem 3, we have ${h}(\mathbf {x}) -1 \in \mathcal {M}_{\mathbf {x},\mathbf {y}}(f_1(\mathbf {x},\mathbf {y}),\ldots , f_m(\mathbf {x},\mathbf {y})).$ Similarly, $-{h}(\mathbf {x}) -1 \in \mathcal {M}_{\mathbf {x},\mathbf {z}}(g_1(\mathbf {x},\mathbf {z}),\ldots , g_n(\mathbf {x},\mathbf {z})).$ That is, there exist $m+n+2$ SOS polynomials $u_i,v_j$ satisfying the following semi-definite constraints:

$$\begin{aligned} h(\mathbf {x})-1=\sum _{i=1}^m u_if_i+u_{m+1}, \quad -h(\mathbf {x})-1=\sum _{j=1}^nv_jg_j+ v_{n+1}. \end{aligned}$$

   $$\square $$

According to Theorem 5, the problem of finding $h(\mathbf {x})\in \mathbb {R}[\mathbf {x}]$ solving Problem 1 can be equivalently reformulated as the problem of searching for SOS polynomials $u_1(\mathbf {x},\mathbf {y}),\ldots ,u_{m}(\mathbf {x},\mathbf {y})$, $v_1(\mathbf {x},\mathbf {z}),\ldots ,v_{n}(\mathbf {x},\mathbf {z})$ and a polynomial $h(\mathbf {x})$ with appropriate degrees such that

9 $$\begin{aligned} \left\{ \begin{aligned}&h(\mathbf {x})-1-\sum _{i=1}^m u_if_i \in \sum \mathbb {R}[\mathbf {x},\mathbf {y}]^2,\\&-h(\mathbf {x})-1-\sum _{j=1}^nv_jg_j\in \sum \mathbb {R}[\mathbf {x},\mathbf {z}]^2,\\&u_i \in \sum \mathbb {R}[\mathbf {x},\mathbf {y}]^2, i=1,\ldots ,m,\\&v_j \in \sum \mathbb {R}[\mathbf {x},\mathbf {z}]^2, j=1,\ldots ,n. \end{aligned} \right. \end{aligned}$$

(9) is SOS constraints over SOS multipliers $u_1(\mathbf {x},\mathbf {y}),\ldots ,u_{m}(\mathbf {x},\mathbf {y})$, $v_1(\mathbf {x},\mathbf {z}), \ldots , v_{n}(\mathbf {x},\mathbf {z})$, polynomial $h(\mathbf {x})$, which is convex and could be solved by many existing semi-definite programming solvers such as the optimization library AiSat [7] built on CSDP [4]. Therefore, according to Theorem 5, $h(\mathbf {x})>0$ is an interpolant for $\phi$ and $\psi$, which is formulated in Theorem 6.

### Theorem 6

**(Soundness).** Suppose that $\phi (\mathbf {x},\mathbf {y})$ and $\psi (\mathbf {x},\mathbf {z})$ are defined as in Problem 1, and $h(\mathbf {x})$ is a feasible solution to (9), then $h(\mathbf {x})$ solves Problem 1, i.e. $h(\mathbf {x})>0$ is an interpolant for $\phi$ and $\psi$.

Moreover, we have the following completeness theorem stating that if the degrees of $h(\mathbf {x})\in \mathbb {R}[\mathbf {x}]$ and $u_i(\mathbf {x},\mathbf {y})\in \sum \mathbb {R}[\mathbf {x},\mathbf {y}]^2,v_j(\mathbf {x},\mathbf {z})\in \sum \mathbb {R}[\mathbf {x},\mathbf {z}]^2$, $i=1,\ldots ,m$, $j=1,\ldots ,n$, are large enough, $h(\mathbf {x})$ can be synthesized definitely via solving (9).

### Theorem 7

**(Completeness).** For Problem 1, there must be polynomials $u_i(\mathbf {x},\mathbf {y})\in \mathbb {R}_N[\mathbf {x},\mathbf {y}]$ $(i=1,\ldots ,m)$, $v_j(\mathbf {x},\mathbf {z})\in \mathbb {R}_N[\mathbf {x},\mathbf {z}]$ $(j=1,\ldots ,n)$ and $h(\mathbf {x})\in \mathbb {R}_N[\mathbf {x}]$ satisfying (11) for some positive integer *N*, where $\mathbb {R}_k[\cdot ]$ stands for the family of polynomials of degree no more than *k*.

### Proof

This is an immediate result of Theorem 5.    $\square$

### Example 2

Consider two contradictory formulas $\phi$ and $\psi$ defined by

$$\begin{aligned} f_1(x,y,z,a_1,b_1,c_1,d_1)\ge 0 \wedge f_2(x,y,z,a_1,b_1,c_1,d_1)\ge 0 \wedge f_3(x,y,z,a_1,b_1,c_1,d_1)\ge 0, \\ g_1(x,y,z,a_2,b_2,c_2,d_2)\ge 0 \wedge g_2(x,y,z,a_2,b_2,c_2,d_2)\ge 0 \wedge g_3(x,y,z,a_2,b_2,c_2,d_2)\ge 0, \end{aligned}$$

respectively, where 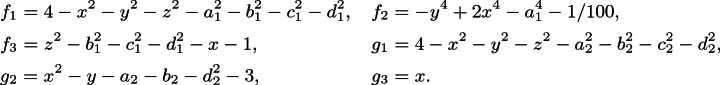 It is easy to observe that $\phi$ and $\psi$ satisfy the conditions in Problem 1. Since there are local variables in $\phi$ and $\psi$ and the degree of $f_2$ is 4, the interpolant generation methods in [7] and [10] are not applicable. We get a concrete $\mathrm{SDP}$ problem of the form (9) by setting the degree of the polynomial *h*(*x*, *y*, *z*) in (9) to be 2. Using the MATLAB package YALMIP [23] and Mosek [28], we obtain

$$\begin{aligned} h(x,y,z)=&-416.7204-914.7840x+472.6184y +199.8985x^2+ 190.2252y^2 \\&+690.4208z^2- 187.1592xy. \end{aligned}$$

**Fig. 2.**
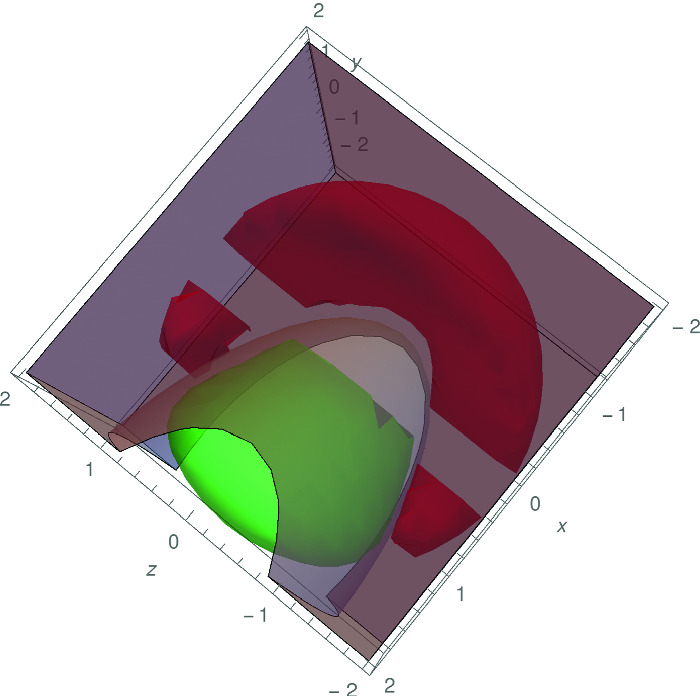
Example 2. (Red region: $P_{x,y,z}(\phi (x,y,z,a_1,b_1,c_1,d_1))$; green region: $P_{x,y,z}(\psi (x,y,z,a_2,b_2,c_2,d_2))$; gray region: $\{(x,y,z)\mid h(x,y,z)>0\}$.) (Color figure online)

Pictorially, we plot $$P_{x,y,z}(\phi (x,y,z,a_1,b_1,c_1,d_1))$$, $$P_{x,y,z}(\psi (x,y,z,a_2,b_2,c_2,$$ $$d_2))$$ and $$\{(x,y,z)\mid h(x,y,z)>0\}$$ in Fig. 2. It is evident that *h*(*x*, *y*, *z*) as presented above for $$d_h=2$$ is a real interpolant for $$\phi (x,y,z,a,b,c,d)$$ and $$\psi (x,y,z,a,b,c,d)$$.

## Avoidance of the Unsoundness Due to Numerical Error in SDP

In this section, we discuss how to avoid the unsoundness of our approach caused by numerical error in SDP based on the work in [32].

A square matrix *A* is *positive semidefinite* if *A* is real symmetric and all its eigenvalues are nonnegative, denote by $A \succeq 0$.

In order to solve formula (9) to obtain $h(\mathbf {x})$, we first need to fix a degree bound of $u_i$, $v_j$ and *h*, say 2*d*, $d\in \mathbb {N}$. It is well-known that any $u(\mathbf {x}) \in \sum \mathbb {R}[\mathbf {x}]^2$ with degree 2*d* can be represented by

10 $$\begin{aligned} u(\mathbf {x}) \equiv E_d(\mathbf {x})^T C_u E_d(\mathbf {x}), \end{aligned}$$

where $$C_u\in \mathbb {R}^{\left( {\begin{array}{c}r+d\\ d\end{array}}\right) \times \left( {\begin{array}{c}r+d\\ d\end{array}}\right) }$$ with $C_u\succeq 0$, $E_d(\mathbf {x})$ is a column vector with all monomials in $\mathbf {x}$, whose total degree is not greater than *d*, and $E_d(\mathbf {x})^T$ stands for the transposition of $E_d(\mathbf {x})$. Equaling the corresponding coefficient of each monomial whose degree is less than or equal to 2*d* at the two sides of (10), we can get a linear equation system as

11 $$\begin{aligned} \mathtt {tr}(A_{u,k}C_u)=b_{u,k},~ k=1,\ldots ,K_u, \end{aligned}$$

where $$A_{u,k}\in \mathbb {R}^{\left( {\begin{array}{c}r+d\\ d\end{array}}\right) \times \left( {\begin{array}{c}r+d\\ d\end{array}}\right) }$$ is constant matrix, $b_{u,k}\in \mathbb {R}$ is constant, $\mathtt {tr}(A)$ stands for the trace of matrix *A*. Thus, searching for $u_i$, $v_j$ and *h* satisfying (9) can be reduced to the following SDP problem:

12 $$\begin{aligned} \begin{aligned}&\ \mathtt {find} :~ C_{u_1},\ldots ,C_{u_m},C_{v_1},\ldots ,C_{v_n},C_{h},\\&\quad \,\,\,\mathtt {s.t.}~\mathtt {tr}(A_{u_i,k}C_{u_i})=b_{u_i,k},~i=1,\ldots ,m, k=1,\ldots ,K_{u_i},\\&\qquad \quad \,\,\,\mathtt {tr}(A_{v_j,k}C_{v_j})=b_{v_j,k},~j=1,\ldots ,n, k=1,\ldots ,K_{v_j},\\&\qquad \quad \,\,\,\mathtt {tr}(A_{h,k}C_h)=b_{h,k},~ k=1,\ldots ,K_h,\\&\mathtt {diag}(C_{u_1},\ldots ,C_{u_m},C_{v_1},\ldots ,C_{v_n},C_{h-1-uf},C_{-h-1-vg})\succeq 0, \end{aligned} \end{aligned}$$

where $C_{h-1-uf}$ is the matrix corresponding to polynomial $h-1-\sum _{i=1}^m u_if_i$, which is a linear combination of $C_{u_1}$, ..., $C_{u_m}$ and $C_h$; similarly, $C_{-h-1-vg}$ is the matrix corresponding to polynomial $-h-1-\sum _{j=1}^n v_jg_j$, which is a linear combination of $C_{v_1}$, ..., $C_{v_n}$ and $C_h$; and $\mathtt {diag}(C_1,\ldots ,C_k)$ is a block-diagonal matrix of $C_1, \ldots , C_k$.

Let *D* be the dimension of $C=\mathtt {diag}(C_{u_1},\ldots ,C_{-h-1-vg})$, i.e., $\mathtt {diag}(C_{u_1}, \ldots , C_{-h-1-vg})\in \mathbb {R}^{D\times D}$ and $\widehat{C}$ be the approximate solution to (12) returned by calling a numerical SDP solver, the following theorem is proved in [32].

### Theorem 8

**(** [32]**, Theorem 3).**$C\succeq 0$ if there exists $\widetilde{C}\in \mathbb {F}^{D\times D}$ such that the following conditions hold: 1. $\widetilde{C}_{ij}=C_{ij}$, for any $i\ne j$; 2. $\widetilde{C}_{ii}\le C_{ii}-\alpha$, for any *i*; and 3. the Cholesky algorithm implemented in floating-point arithmetic can conclude that $\widetilde{C}$ is positive semi-definite, where $\mathbb {F}$ is a floating-point format, $\alpha =\frac{(D+1)\kappa }{1-(2D+2)\kappa }\mathtt {tr}(C)+4(D+1)(2(D+2)+\max _i\{C_{ii}\} ) \eta$, in which $\kappa$ is the unit roundoff of $\mathbb {F}$ and $\eta$ is the underflow unit of $\mathbb {F}$.

### Corollary 2

Let $\widetilde{C} \in \mathbb {F}^{D\times D}$. Suppose that $\frac{(D+1)D \kappa }{1-(2D+2)\kappa }+4(D+1)\eta \le \frac{1}{2}$, $\beta = \frac{(D+1)\kappa }{1-(2D+2)\kappa }\mathtt {tr}(\widetilde{C})+4(D+1)(2(D+2)+\max _i\{\widetilde{C}_{ii}\}) \eta >0$, where $\mathbb {F}$ is a floating-point format. Then $\widetilde{C}+2\beta I\succeq 0$ if the Cholesky algorithm based on floating-point arithmetic succeeds on $\widetilde{C}$, i.e., concludes that $\widetilde{C}$ is positive semi-definite.

According to Remark 5 in [32], for IEEE 754 binary64 format with rounding to nearest, $\kappa =2^{-53}(\simeq 10^{-16})$ and $\eta =2^{-1075}(\simeq 10^{-323})$. In this case, the order of magnitude of $\beta$ is $10^{-10}$ and $\frac{(D+1)D \kappa }{1-(2D+2)\kappa }+4(D+1)\eta$ is $10^{-13}$, much less than $\frac{1}{2}$. Obviously, $\beta$ becomes smaller when the length of binary format becomes longer. W.l.o.g., we suppose that the Cholesky algorithm succeed in computing $\widehat{C}$ the solution of (12), which is reasonable as if an SDP solver returns a solution $\widehat{C}$, then $\widehat{C}$ should be considered to be positive semi-definite in the sense of numeric computation.

So, by Corollary 2, we have $\widehat{C}+2\beta I\succeq 0$ holds, where *I* is the identity matrix with the corresponding dimension. Then we have

$$\begin{aligned} \mathtt {diag}(\widehat{C}_{u_1}, \ldots , \widehat{C}_{u_m}, \widehat{C}_{v_1}, \ldots , \widehat{C}_{v_n}, \widehat{C}_{h-1-uf}, \widehat{C}_{-h-1-vg}) +2\beta I\succeq 0. \end{aligned}$$

Let $\epsilon =\max _{p\in P,1\le i\le K_p} |\mathtt {tr}(A_{p,i}\widehat{C}_p)-b_{p,i}|$, where $P=\{u_1,\ldots ,u_m,v_1,\ldots ,v_n,h\}$, which can be regarded as the tolerance of the SDP solver. Since $|\mathtt {tr}(A_{p,i}C_p)-b_{p,i}|$ is the error term for each monomial of *p*, i.e., $\epsilon$ can be considered as the error bound on the coefficients of polynomials $u_i$, $v_j$ and *h*, for any polynomial $\hat{u_i}$ ( $\hat{v_j}$ and $\hat{h}$), computed from (11) by replacing $C_u$ with the corresponding $\widehat{C_u}$, there exists a corresponding remainder term $R_{u_i}$ (resp. $R_{v_j}$ and $R_h$) with degree not greater than 2*d*, whose coefficients are bounded by $\epsilon$. Hence, we have

13 $$\begin{aligned} \begin{aligned}&\widehat{u_i}+R_{u_i}+2\beta E_d(\mathbf {x},\mathbf {y})^T E_d(\mathbf {x},\mathbf {y})\in \sum \mathbb {R}[\mathbf {x},\mathbf {y}]^2, i=1,\ldots ,m, \\&\widehat{v_j}+R_{v_j}+2\beta E_d(\mathbf {x},\mathbf {z})^T E_d(\mathbf {x},\mathbf {z}) \in \sum \mathbb {R}[\mathbf {x},\mathbf {z}]^2, j=1,\ldots ,n, \\&\widehat{h}+R_{h}-1-\sum _{i=1}^m (\widehat{u_i}+R'_{u_i}) f_i+2\beta E_d(\mathbf {x},\mathbf {y})^T E_d(\mathbf {x},\mathbf {y}) \in \sum \mathbb {R}[\mathbf {x},\mathbf {y}]^2, \\ -&\widehat{h}+R'_{h}-1-\sum _{j=1}^m (\widehat{v_j}+R'_{v_j}) g_j+2\beta E_d(\mathbf {x},\mathbf {z})^T E_d(\mathbf {x},\mathbf {z}) \in \sum \mathbb {R}[\mathbf {x},\mathbf {z}]^2. \end{aligned} \end{aligned}$$

Now, in order to avoid unsoundness of our approach caused by the numerical issue due to SDP, we have to prove

14 $$\begin{aligned} f_1\ge 0\wedge \cdots \wedge f_m\ge 0 \Rightarrow \widehat{h}>0,\end{aligned}$$

15 $$\begin{aligned} g_1\ge 0\wedge \cdots \wedge g_n\ge 0 \Rightarrow \widehat{h}<0. \end{aligned}$$

Regarding (14), let $R_{2d,\mathbf {x}}$ be a polynomial in $\mathbb {R}[|\mathbf {x}|]$, whose total degree is 2*d*, and all coefficients are 1, e.g., $R_{2,x,y}=1+|x|+|y|+|x^2|+|xy|+|y^2|$. Since $S=\{(\mathbf {x},\mathbf {y}) \mid f_1\ge 0\wedge \cdots \wedge f_m\ge 0\}$ is a compact set, then for any polynomial $p\in \mathbb {R}[\mathbf {x},\mathbf {y}]$, |*p*| is bounded on *S*. Let $M_1$ be an upper bound of $R_{2d,\mathbf {x},\mathbf {y}}$ on *S*, $M_2$ an upper bound of $E_d(\mathbf {x},\mathbf {y})^T E_d(\mathbf {x},\mathbf {y})$, and $M_{f_i}$ an upper bound of $f_i$ on *S*. Then, $|R_{u_i}|$, $|R'_{u_i}|$ and $|R_{h}|$ are bounded by $\epsilon M_1$. Let $E_{\mathbf {x}\mathbf {y}}=E_d(\mathbf {x},\mathbf {y})^T E_d(\mathbf {x},\mathbf {y})$. So for any $(\mathbf {x}_0,\mathbf {y}_0)\in S$, considering the polynomials below at $(\mathbf {x}_0,\mathbf {y}_0)\in S$, by the first and third line in (13),

$$\begin{aligned} \widehat{h}&\ge \, 1-R_{h}+\sum _{i=1}^m (\widehat{u_i}+R'_{u_i}) f_i-2\beta E_{\mathbf {x}\mathbf {y}}\\&\ge \, 1- \epsilon M_1+\sum _{i=1}^m(\widehat{u_i}+R_{u_i}+2\beta E_{\mathbf {x}\mathbf {y}}+R'_{u_i}-R_{u_i}-2\beta E_{xy}) f_i-2\beta M_2 \\&=\, 1- \epsilon M_1-2\beta M_2 +\sum _{i=1}^m(\widehat{u_i}+R_{u_i}+2\beta E_{\mathbf {x}\mathbf {y}}) f_i+\sum _{i=1}^m(R'_{u_i}-R_{u_i}-2\beta E_{\mathbf {x}\mathbf {y}}) f_i \\&\ge \, 1- \epsilon M_1-2\beta M_2 +0-\sum _{i=1}^m (\epsilon M_1+\epsilon M_1+2\beta M_2)M_{f_i} \\&=\, 1-(2\sum _{i=1}^m M_{f_i}+1)M_1 \epsilon - 2(\sum _{i=1}^m M_{f_i}+1)M_2 \beta . \end{aligned}$$

Whence,

$$\begin{aligned} f_1\ge 0\wedge \cdots \wedge f_m\ge 0 \Rightarrow \widehat{h}\ge 1-(2\sum _{i=1}^m M_{f_i}+1)M_1 \epsilon - 2(\sum _{i=1}^m M_{f_i}+1)M_2 \beta . \end{aligned}$$

Let $S'=\{(\mathbf {x},\mathbf {z}) \mid g_1\ge 0\wedge \cdots \wedge g_n\ge 0\}$, $M_3$ be an upper bound of $R_{2d,\mathbf {x},\mathbf {z}}$ on $S'$, $M_4$ an upper bound of $E_d(\mathbf {x},\mathbf {z})^T E_d(\mathbf {x},\mathbf {z})$ on $S'$, and $M_{g_j}$ an upper bound of $g_j$ on $S'$. Similarly, it follows

$$\begin{aligned}&g_1\ge 0\wedge \cdots \wedge g_n\ge 0 \Rightarrow&-\widehat{h}\ge 1-(2\sum _{j=1}^n M_{g_j}+1)M_3 \epsilon - 2(\sum _{j=1}^n M_{g_j}+1)M_4 \beta . \end{aligned}$$

So, the following proposition is immediately.

### Proposition 2

There exist two positive constants $\gamma _1$ and $\gamma _2$ such that

16 $$\begin{aligned} f_1\ge 0\wedge \cdots \wedge f_m\ge 0 \Rightarrow \widehat{h}\ge 1-\gamma _1 \epsilon - \gamma _2 \beta , \end{aligned}$$

17 $$\begin{aligned} g_1\ge 0\wedge \cdots \wedge g_n\ge 0 \Rightarrow -\widehat{h}\ge 1-\gamma _1 \epsilon - \gamma _2 \beta . \end{aligned}$$

Since $\epsilon$ and $\beta$ heavily rely on the numerical tolerance and the floating point representation, it is easy to see that $\epsilon$ and $\beta$ become small enough with $\gamma _1 \epsilon <\frac{1}{2}$ and $\gamma _2 \beta <\frac{1}{2}$, if the numerical tolerance is small enough and the length of the floating point representation is long enough. This implies

$$\begin{aligned} f_1\ge 0\wedge \cdots \wedge f_m\ge 0 \Rightarrow \widehat{h}>0, \quad g_1\ge 0\wedge \cdots \wedge g_n\ge 0 \Rightarrow -\widehat{h}>0. \end{aligned}$$

If so, any numerical result $\widehat{h}>0$ returned by calling an SDP solver to (12) is guaranteed to be a real interpolant for $\phi$ and $\psi$, i.e., a correct solution to Problem 1.

### Example 3

Consider the numerical result for Example 2 in Sect. 4. Let $M_{f_1}$, $M_{f_2}$, $M_{f_3}$, $M_{g_1}$, $M_{g_2}$, $M_{g_3}$, $M_1$, $M_2$, $M_3$, $M_4$ are defined as above. It is easy to see that

$$\begin{aligned} f_1\ge 0\Rightarrow&|x|\le 2\wedge |y|\le 2\wedge |z|\le 2\wedge |a_1|\le 2\wedge |b_1|\le 2 \wedge |c_1|\le 2\wedge |d_1|\le 2. \end{aligned}$$

Then, by simple calculations, we obtain $M_{f_1}=4, M_{f_2}=32, M_{f_3}=3, M_1=83, M_2=29.$ Thus,

$$(2\sum _{i=1}^m M_{f_i}+1)M_1=6557,\quad 2(\sum _{i=1}^m M_{f_i}+1)M_2=2320.$$

Also, since

$$\begin{aligned} g_1\ge 0\Rightarrow&|x|\le 2\wedge |y|\le 2\wedge |z|\le 2\wedge |a_2|\le 2\wedge |b_2|\le 2 \wedge |c_2|\le 2\wedge |d_2|\le 2, \end{aligned}$$

we obtain $M_{g_1}=4, M_{g_2}=7, M_{g_3}=2, M_3=83, M_4=29.$ Thus,

$$(2\sum _{i=1}^m M_{g_i}+1)M_3=2241,\quad 2(\sum _{i=1}^m M_{g_i}+1)M_4=812.$$

Consequently, we have $\gamma _1=6557$ and $\gamma _2=2320$ in Proposition 2.

Due to the fact that the default error tolerance is $10^{-8}$ in the SDP solver Mosek and *h* is rounding to 4 decimal places, we have $\epsilon =\frac{10^{-4}}{2}$. In addition, as the absolute value of each element in $\widehat{C}$ is less than $10^3$, and the dimension of *D* is less than $10^3$, we obtain

$$\begin{aligned} \beta&= \frac{(D+1)\kappa }{1-(2D+2)\kappa }\mathtt {tr}(\widetilde{C}) +4(D+1)(2(D+2)+\max _i(\widetilde{C}_{ii})) \eta \le 10^{-6}. \end{aligned}$$

Consequently, $\gamma _1 \epsilon \le 6557 \cdot \frac{10^{-4}}{2} <\frac{1}{2}$, $\gamma _2 \beta \le 2320 \cdot 10^{-6} < \frac{1}{2}$, which imply that $h(x,y,z)>0$ presented in Example 2 is indeed a real interpolant.

### Remark 1

Besides, the result could be verified by the following symbolic computation procedure instead: computing $P_\mathbf {x}(\phi )$ and $P_\mathbf {x}(\psi )$ first by some symbolic tools, such as Redlog [8] which is a package that extends the computer algebra system REDUCE to a computer logic system; then verifying $\mathbf {x}\in P_\mathbf {x}(\phi )\Rightarrow h(\mathbf {x})>0$ and $\mathbf {x}\in P_\mathbf {x}(\psi )\Rightarrow h(\mathbf {x})<0.$ For this example, $P_{x,y,z}(\phi )$ and $P_{x,y,z}(\psi )$ obtained by Redlog are too complicated and therefore not presented here. The symbolic computation can verify that *h*(*x*, *y*, *z*) in this example is exactly an interpolant, which confirms our conclusion. Alternatively, we can also solve the SDP in (9) using a SDP solver with infinite precision [15], and obtain an exact result. But this only works for problems with small size because a SDP solver with infinite precision is essentially based on symbolic computation as commented in [15].

## Generalizing to General Polynomial Formulas

### Problem 2

Let $\phi (\mathbf {x},\mathbf {y})$ and $\psi (\mathbf {x},\mathbf {z})$ be two polynomial formulas defined as follows,

$$\begin{aligned} \phi (\mathbf {x},\mathbf {y}) : \bigvee _{i=1}^m \phi _i, ~ \phi _i=\bigwedge _{k=1}^{K_i}f_{i,k}(\mathbf {x},\mathbf {y}) \ge 0; \quad \psi (\mathbf {x},\mathbf {z}) : \bigvee _{j=1}^n \psi _j, ~ \psi _j=\bigwedge _{s=1}^{S_j}g_{j,s}(\mathbf {x},\mathbf {z}) \ge 0 , \end{aligned}$$

where all $f_{i,k}$ and $g_{j,s}$ are polynomials. Suppose $\phi \wedge \psi \models \bot$, and for $i=1,\ldots ,m$, $j=1,\ldots ,n$, $\{(\mathbf {x},\mathbf {y})\mid \phi _i(\mathbf {x},\mathbf {y})\}$ and $\{(\mathbf {x},\mathbf {z})\mid \psi _j(\mathbf {x},\mathbf {z})\}$ are all semi-algebraic sets of the Archimedean form. Find a polynomial $h(\mathbf {x})$ such that $h(\mathbf {x})>0$ is an interpolant for $\phi$ and $\psi$.

### Theorem 9

For Problem 2, there exists a polynomial $h(\mathbf {x})$ satisfying

$$\forall \mathbf {x}\in P_{\mathbf {x}}(\phi (\mathbf {x},\mathbf {y})). h(\mathbf {x}) > 0, \quad \forall \mathbf {x}\in P_{\mathbf {x}}(\psi (\mathbf {x},\mathbf {z})). h(\mathbf {x}) < 0.$$

### Proof

We just need to prove that Lemma 1 holds for Problem 2 as well. Since $\{(\mathbf {x},\mathbf {y})\mid \phi _i(\mathbf {x},\mathbf {y})\}$ and $\{(\mathbf {x},\mathbf {z})\mid \psi _j(\mathbf {x},\mathbf {z})\}$ are all semi-algebraic sets of the Archimedean form, then $\{(\mathbf {x},\mathbf {y})\mid \phi (\mathbf {x},\mathbf {y})\}$ and $\{(\mathbf {x},\mathbf {z})\mid \psi (\mathbf {x},\mathbf {z})\}$ both are compact. See $\{(\mathbf {x},\mathbf {y})\mid \phi (\mathbf {x},\mathbf {y})\}$ or $\{(\mathbf {x},\mathbf {z})\mid \psi (\mathbf {x},\mathbf {z})\}$ as *S* in the proof of Lemma 1, then Lemma 1 holds for Problem 2. Thus, the rest of proof is same as that forTheorem 2.    $\square$

### Corollary 3

Let $\phi (\mathbf {x},\mathbf {y})$ and $\psi (\mathbf {x},\mathbf {z})$ be defined as in Problem 2. There must exist a polynomial $h(\mathbf {x})$ such that $h(\mathbf {x})>0$ is an interpolant for $\phi$ and $\psi$.

### Theorem 10

Let $\phi (\mathbf {x},\mathbf {y})$ and $\psi (\mathbf {x},\mathbf {z})$ be defined as in Problem 2. Then there exists a polynomial $h(\mathbf {x})$ and $\sum _{i=1}^m(K_i+1)+\sum _{j=1}^n (S_j+1)$ sum of squares polynomials $u_{i,k}(\mathbf {x},\mathbf {y})$ $(i=1,\ldots ,m$, $k=1,\ldots ,K_i+1)$, $v_{j,s}(\mathbf {x},\mathbf {z})$ $(j=1,\ldots ,n$, $s=1,\ldots ,S_j)$ satisfying the following semi-definite constraints such that $h(\mathbf {x})>0$ is an interpolant for $\phi (\mathbf {x},\mathbf {y})$ and $\psi (\mathbf {x},\mathbf {z})$:

18 $$\begin{aligned}&h-1=\sum _{k=1}^{K_i} u_{i,k}f_{i,k}+u_{i,K_i+1},\quad i=1,\ldots , m;\end{aligned}$$

19 $$\begin{aligned}&-h-1=\sum _{s=1}^{S_j} v_{j,s}g_{j,s}+ v_{j,S_j+1}, \quad j=1,\ldots , n. \end{aligned}$$

### Proof

By the property of Archimedean, the proof is same as that for Theorem 5.    $\square$

Similarly, Problem 2 can be equivalently reformulated as the problem of searching for sum of squares polynomials satisfying

20 $$\begin{aligned} \left\{ \begin{aligned}&h(\mathbf {x})-1-\sum _{k=1}^{K_i} u_{i,k}f_{i,k} \in \sum \mathbb {R}[\mathbf {x},\mathbf {y}]^2, i=1,\ldots ,m;\\&-h(\mathbf {x})-1-\sum _{s=1}^{S_j} v_{j,s}g_{j,s}\in \sum \mathbb {R}[\mathbf {x},\mathbf {z}]^2, j=1,\ldots ,n;\\&u_{i,k} \in \sum \mathbb {R}[\mathbf {x},\mathbf {y}]^2, i=1,\ldots ,m, k=1,\ldots , K_i;\\&v_{j,s} \in \sum \mathbb {R}[\mathbf {x},\mathbf {z}]^2, j=1,\ldots ,n, s=1,\ldots , S_j. \end{aligned} \right. \end{aligned}$$

### Example 4

Consider

$$\begin{aligned} \phi (x,y,a_1,a_2,b_1,b_2): (f_1\ge 0\wedge f_2\ge 0)\vee (f_3\ge 0 \wedge f_4\ge 0),\\ \psi (x,y,c_1,c_2,d_1,d_2): (g_1\ge 0\wedge g_2\ge 0)\vee (g_3\ge 0 \wedge g_4\ge 0), \end{aligned}$$

where

$$\begin{aligned} f_1=16-(x+y-4)^2-16(x-y)^2-a_1^2,&f_2=x+y-a_2^2-(2-a_2)^2,\\ f_3=16-(x+y+4)^2-16(x-y)^2-b_1^2,&f_4=-x-y-b_2^2-(2-b_2)^2,\\ g_1=16-16(x+y)^2-(x-y+4)^2-c_1^2,&g_2=y-x-c_2^2-(1-c_2)^2,\\ g_3=16-16(x+y)^2-(x-y-4)^2-d_1^2,&g_4=x-y-d_2^2-(1-d_2)^2. \end{aligned}$$

We get a concrete $\mathrm{SDP}$ problem of the form (20) by setting the degree of *h*(*x*, *y*) in (20) to be 2. Using the MATLAB package YALMIP and Mosek, we obtain

$$\begin{aligned} h(x,y)=-2.3238+0.6957x^2+0.6957y^2+7.6524xy. \end{aligned}$$

**Fig. 3.**
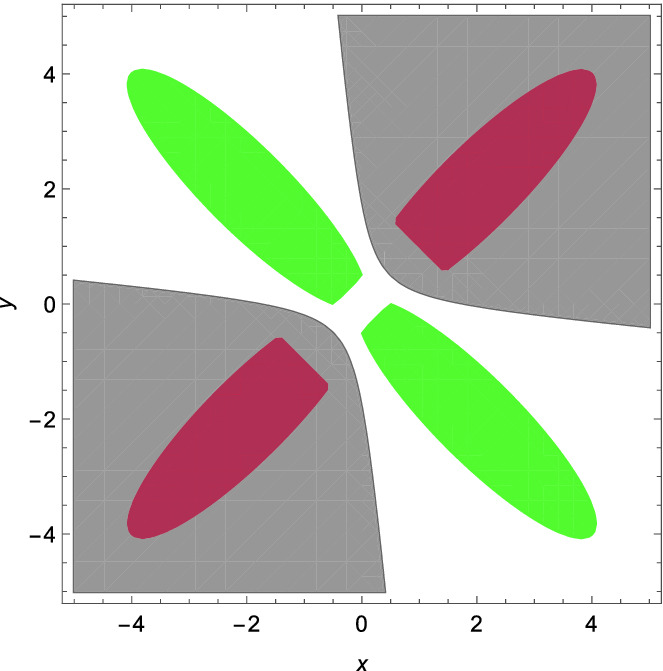
Example 4. (Red region:   $P_{x,y}(\phi (x,y,a_1,a_2,b_1,b_2))$; green region:  $P_{x,y}(\psi (x,y,c_1,c_2,d_1,d_2))$; gray region:  $\{(x,y)\mid h(x,y)>0\}$.) (Color figure online)

The result is plotted in Fig. 3, and can be verified either by numerical error analysis as in Example 2 or by a symbolic procedure like REDUCE as described in Remark 1.

**Fig. 4.**
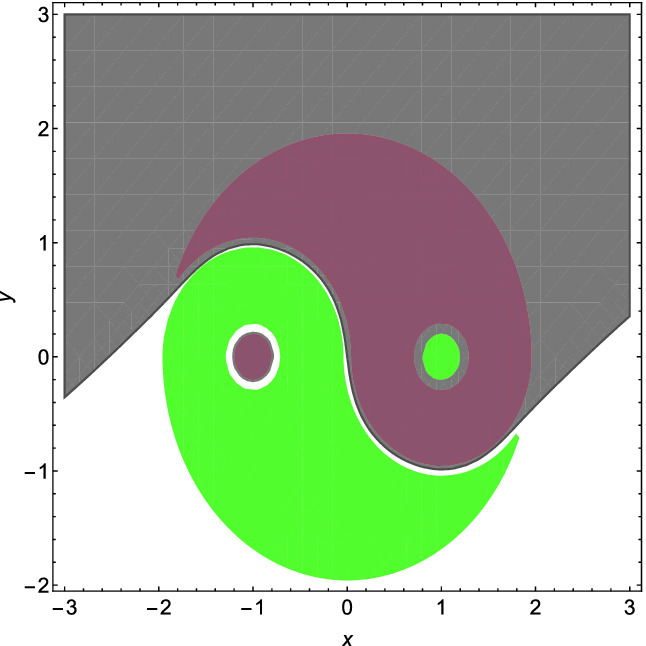
Example 5. (Red region: $P_{x,y}(\phi (x,y))$; green region: $P_{x,y}(\psi (x,y))$; gray region: $\{(x,y)\mid h(x,y)>0\}$.) (Color figure online)

### Example 5

*(Ultimate).* Consider the following example taken from [5], which is a challenging benchmark to existing approaches for nonlinear interpolant generation.

$$\begin{aligned}&\phi = (f_1\ge 0 \wedge f_2\ge 0 \vee f_3\ge 0) \wedge f_4\ge 0 \wedge f_5\ge 0 \vee f_6\ge 0, \\&\psi = (g_1\ge 0 \wedge g_2\ge 0 \vee g_3\ge 0) \wedge g_4\ge 0 \wedge g_5\ge 0 \vee g_6\ge 0, \end{aligned}$$

where

$$ \begin{array}{lcl} f_1 = 3.8025-x^2-y^2, &{} \quad \quad &{} f_2 = y, \\ f_3 = 0.9025-(x-1)^2-y^2, &{} &{} f_4 = (x-1)^2+y^2-0.09, \\ f_5 = (x+1)^2+y^2-1.1025, &{} &{} f_6 = 0.04-(x+1)^2-y^2, \\ g_1 = 3.8025-x^2-y^2, &{} &{} g_2 = -y, \\ g_3 = 0.9025-(x+1)^2-y^2, &{} &{} g_4 = (x+1)^2+y^2-0.09, \\ g_5 = (x-1)^2+y^2-1.1025, &{} &{} g_6 = 0.04-(x-1)^2-y^2. \end{array} $$

We first convert $\phi$ and $\psi$ to the disjunction normal form as:

$$\begin{aligned} \phi =&(f_1\ge 0 \wedge f_2\ge 0 \wedge f_4\ge 0 \wedge f_5 \ge 0) \vee (f_3\ge 0\wedge f_4\ge 0 \wedge f_5\ge 0) \vee (f_6\ge 0), \\ \psi =&(g_1\ge 0 \wedge g_2\ge 0 \wedge g_4\ge 0 \wedge g_5\ge 0) \vee (g_3\ge 0 \wedge g_4\ge 0 \wedge g_5\ge 0) \vee (g_6\ge 0). \end{aligned}$$

We get a concrete $\mathrm{SDP}$ problem of the form (20) by setting the degree of *h*(*x*, *y*) in (20) to be 7. Using the MATLAB package YALMIP and Mosek, keeping the decimal to four, we obtain

$$\begin{aligned} h&(x,y)=1297.5980x+191.3260y-3172.9653x^3+196.5763x^2y +2168.1739xy^2\\&+\,1045.7373y^3 +1885.8986x^5-1009.6275x^4y +3205.3793x^3y^2-1403.5431x^2y^3 \\&+\, 1842.0669xy^4 +1075.2003y^5-222.0698x^7+ 547.9542x^6y-704.7474x^5y^2\\&+\,1724.7008x^4y^3-728.2229x^3y^4+1775.7548x^2y^5-413.3771xy^6+1210.2617y^7. \end{aligned}$$

The result is plotted in Fig. 4, and can be verified either by numerical error analysis as in Example 2 or by a symbolic procedure like REDUCE as described in Remark 1.

## Application to Invariant Generation

In this section, as an application, we sketch how to apply our approach to invariant generation in program verification, the details can be found in [11].

In [22], Lin *et al.* proposed a framework for invariant generation using *weakest precondition*, *strongest postcondition* and *interpolation*, which consists of two procedures, i.e., synthesizing invariants by forward interpolation based on *strongest postcondition* and *interpolant generation*, and by backward interpolation based on *weakest precondition* and *interpolant generation*. In [22], only linear invariants can be synthesized as no powerful approaches are available to synthesize nonlinear interpolants. Obviously, our results can strengthen their framework by allowing to generate nonlinear invariants. For example, we can revise the procedure Squeezing Invariant - Forward in their framework and obtain Algorithm 1.

The major revisions include:

- firstly, we exploit our method to synthesize interpolants see line 4 in Algorithm 1;
- secondly, we add a conditional statement for $A_{i+1}$ at line 7–10 in Algorithm 1 in order to make $A_{i+1}$ to be Archimedean.

The procedure Squeezing Invariant - backward can be revised similarly.
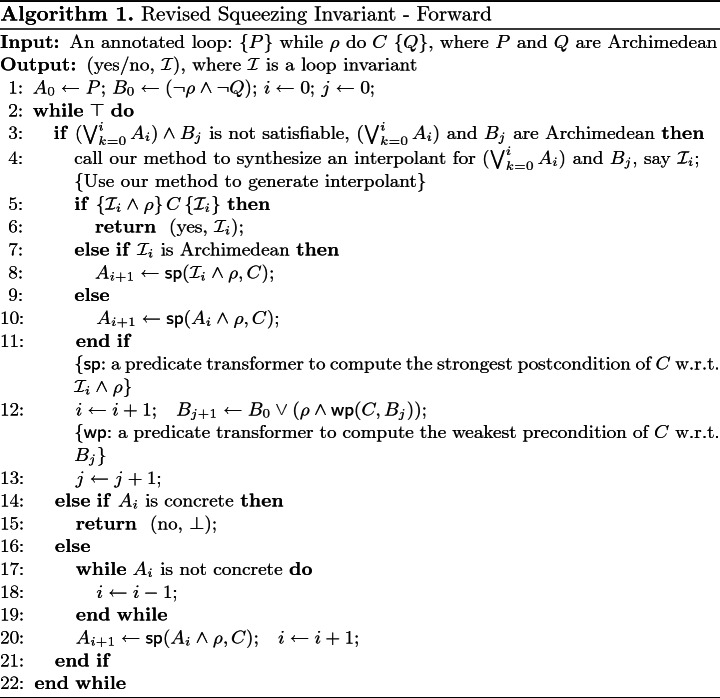


### Example 6

Consider a loop program given in Algorithm 2 for controlling the acceleration of a car adapted from [21]. Suppose we know that $\textit{vc}$ is in [0, 40] at the beginning of the loop, we would like to prove that $\textit{vc}<49.61$ holds after the loop. Since the loop guard is unknown, it means that the loop may terminate after any number of iterations.

We apply Algorithm 1 to the computation of an invariant to ensure that $\textit{vc}<49.61$ holds. Since $\textit{vc}$ is the velocity of car, $0\le \textit{vc}<49.61$ is required to hold in order to maintain safety. Via Algorithm 1, we have $A_0=\{\textit{vc}\mid \textit{vc}(40-\textit{vc})\ge 0\}$ and $B=\{\textit{vc}\mid \textit{vc}<0\}\cup \{ \textit{vc}\mid \textit{vc}\ge 49.61\}$. Here, we replace *B* with $B'=[-2,-1]\cup [49.61,55]$), i.e., $B'=\{\textit{vc}\mid (\textit{vc}+2)(-1-\textit{vc})\ge 0\vee (\textit{vc}-49.61)(55-\textit{vc})\ge 0\}$, in order to make it with Archimedean form.

Firstly, it is evident that $A_0: \textit{vc}(40-\textit{vc})\ge 0$ implies $A_0\wedge B'\models \bot$. By applying our approach, we obtain an interpolant

$$\begin{aligned} \mathcal {I}_0: 1.4378+3.3947*\textit{vc}-0.083*\textit{vc}^2>0 \end{aligned}$$

for $A_0$ and $B'$. It can be verified that $\{\mathcal {I}_0\} \, C \, \{\mathcal {I}_0\}$ (line 5) does not hold, where *C* stands for the loop body.

Secondly, by setting $A_1=sp(\mathcal {I}_0,C)$ (line 8) and re-calling our approach, we obtain an interpolant

$$\mathcal {I}_1: 2.0673+3.0744*\textit{vc}-0.0734*\textit{vc}^2>0$$

for $A_0\cup A_1$ and $B'$. Likewise, it can be verified that $\{\mathcal {I}_1\}\, C \, \{\mathcal {I}_1\}$ (line 5) does not hold.
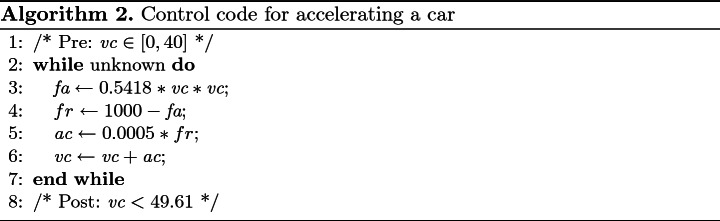


Thirdly, repeating the above procedure again, we obtain an interpolant

$$\mathcal {I}_2: 2.2505+2.7267*\textit{vc}-0.063*\textit{vc}^2>0,$$

and it can be verified that $\{\mathcal {I}_2\} \, C \, \{\mathcal {I}_2\}$ holds, implying that $\mathcal {I}_2$ is an invariant. Moreover, it is trivial to verify that $\mathcal {I}_2\Rightarrow \textit{vc}<49.61$.

Consequently, we have the conclusion that $\mathcal {I}_2$ is an inductive invariant which witnesses the correctness of the loop.

## Conclusion

In this paper we propose a sound and complete method to synthesize Craig interpolants for mutually contradictory polynomial formulas $\phi (\mathbf {x},\mathbf {y})$ and $\psi (\mathbf {x},\mathbf {z})$, with the form $f_1\ge 0\wedge \cdots \wedge f_n\ge 0$, where $f_i$’s are polynomials in $\mathbf {x},\mathbf {y}$ or $\mathbf {x},\mathbf {z}$ and the quadratic module generated by $f_i$’s is Archimedean. The interpolant is generated by solving a semi-definite programming problem, which is a generalization of the method in [7] dealing with mutually contradictory formulas with the same set of variables and the method in [10] dealing with mutually contradictory formulas with concave quadratic polynomial inequalities. As an application, we apply our approach to invariant generation in program verification.

As a future work, we would like to consider interpolant synthesizing for formulas with strict polynomial inequalities. Also, it deserves to consider how to synthesize interpolants for the combination of non-linear formulas and other theories based on our approach and other existing ones, as well as further applications to the verification of programs and hybrid systems.

## Footnotes

1 Alternatively, if we try the formula with the latest version of dReal4, it does not produce any output after 20 h.

2 The mathematical representation of *h* is given in the full version [11].

3 The zero level set of an *n*-variate polynomial $h(\mathbf {x})$ is defined as $\{\mathbf {x}\in \mathbb {R}^n\mid h(\mathbf {x})=0\}.$
